# Seasonal water level changes affect plant diversity and littoral widths at different elevation zones in the Erhai Lake

**DOI:** 10.3389/fpls.2025.1503627

**Published:** 2025-03-20

**Authors:** Feng Zhu, Jing Yuan, Zeying Hou, Xia Guo, Wanxue Liao, Shenglin Yang, Zhaosheng Chu

**Affiliations:** ^1^ State Key Laboratory of Environmental Criteria and Risk Assessment, Chinese Research Academy of Environmental Sciences, Beijing, China; ^2^ College of Water Sciences, Beijing Normal University, Beijing, China; ^3^ National Engineering Laboratory for Lake Pollution Control and Ecological Restoration, Chinese Research Academy of Environmental Sciences, Beijing, China; ^4^ Construction Project Environmental Impact Assessment and Audit Center of Dali Bai Autonomous Prefecture, Dali, Yunnan, China

**Keywords:** littoral widths, plant diversity, soil nutrient, hydrological seasonal changes, niche breadth

## Abstract

The littoral width of lakeshores is crucial for maintaining and promoting plant diversity. However, it remains unclear how changes in seasonal water level affect littoral widths by regulating plant diversity and soil nutrient content. This study selected three elevation ranges in the lakeshore of Erhai: supralittoral, eulittoral, and infralittoral. We explored the effects of hydrological changes on littoral widths and their potential relationships by analyzing seasonal differences in plant communities and soil physicochemical properties during an extremely drought year. Our results indicated that the most significant seasonal differences in diversity indices, biomass, and soil physicochemical properties were observed in the eulittoral, followed by the infralittoral and supralittoral. The niche breadths of perennials was significantly decreased by 44.4% and the width of the eulittoral was significantly decreased by 48.6% during the winter. Generalized Additive Models (GAMs) were applied to analyze the elevation distribution ranges of dominant species. The results revealed that species with monotonically increasing distributions had the widest niche breadths, followed by symmetric unimodal species, while monotonically decreasing species exhibited the narrowest. Structural equation modeling revealed a positive and significant correlation between flooding days and soil water content and pH, and a negative correlation with plant parameters (species number, biomass, and coverage). Moreover, plant parameters showed a significant positive correlation with plant diversity. Importantly, plant diversity and soil nutrients were significantly positively correlated with littoral widths, suggesting their key roles in influencing littoral widths. This study highlights the significant impact of hydrological seasonal changes on the littoral widths of lakeshore zones, providing valuable guidance for managing wetland water levels in response to extreme drought events.

## 1 Introduction

The lakeshore zone is a transitional area between aquatic and terrestrial ecosystems and is a crucial component of the lake ecosystem (Ostendorp, 2004). It serves as a terrestrial-lake ecotone, being a sensitive area susceptible to water level fluctuations, and is also critical for maintaining biodiversity (Wang et al., 2022; Zhang et al., 2015). Lakeshore plants play a crucial role as a primary producer in preventing soil erosion, intercepting pollutants, and providing diverse habitats (Li et al., 2019; Zhang et al., 2021). Seasonal water level changes significantly influence the distribution of lakeshore plants, exhibiting distinct vertical distribution characteristics (Gaberscik et al., 2018; Zheng et al., 2022). Aquatic plants in the infralittoral are adapted to long-term flooding (Baschuk et al., 2012), hygrophytes in the eulittoral are adapted to intermittent flooding (Garssen et al., 2017; Ojdanic et al., 2023), and xerophytes in the supralittoral are adapted to drought conditions (Stroh et al., 2008).

Water level changes are one of the driving factors shaping the landscape patterns of wetlands and a determining factor influencing the spatiotemporal distribution of plant communities (Gathman et al., 2005; Hu et al., 2018). Wetland hydrological processes significantly influence the composition, diversity, distribution width, and area of plant communities (Gaberscik et al., 2018; Wang et al., 2015; You et al., 2015; Zheng et al., 2022). Such as long-time droughts or floods may lead to decreased numbers and diversity of plant species in wetlands, or even the formation of a single dominant species community (Gaberscik et al., 2020; Wassens et al., 2017). Secondly, the frequency and duration of wet-dry alternations significantly affect the growth and physiological characteristics of wetland plants, with frequent alternations increasing plant community diversity (Pollock et al., 1998; Zhang et al., 2022). In addition, fluctuations in the highest and lowest water levels determine the distribution width of plant communities and influence the habitat range of different plant communities (Chapin and Paige, 2013). Water level fluctuations directly or indirectly influence the seed germination and reproductive success of lakeshore plants by affecting soil water and nutrient content (Fu et al., 2018; Zhao et al., 2021). Therefore, wetland plant community structure and growth may vary significantly across elevation zones under different water level gradients.

The response of plant species to environmental gradients reflects adaptive adjustments in their niche (Hutchinson, 1957), which have received extensive attention in wetland research and management. The niche breadth of a species determines its ability to utilize different environmental gradients, thereby influencing its distribution range and competitive ability (Costa et al., 2018). The hydrology, soil, and plants are three important components of wetland ecosystems that interact and influence each other (Feng et al., 2020; Zhang et al., 2022). The constrained spatial extent of lakeshore zones results in the distribution and niche breadth of plants being particularly sensitive to variations in soil physicochemical properties, water levels, and elevation (Lou et al., 2018; Wang et al., 2022; Zheng et al., 2022). For example, larger inter-annual differences in water levels can alter wetland soil moisture and nutrient availability, thereby affecting plant diversity (Shen et al., 2020). Lakeshore zones are ideal habitats for studying changes in plant ecological behavior, as the significant environmental gradients and diverse habitats make the response of plant communities to environmental changes more intuitive and easier to measure (Chen et al., 2020; Duval et al., 2012). However, knowledge is limited on the relationship between plant niche breadth at different elevation ranges in the lakeshore zone and environmental gradients, especially in plateau lakes.

Lake Erhai, the second largest plateau lake in the Yunnan Province of China, serves multiple functions including agricultural irrigation, climate regulation, tourism and water supply (Wang et al., 2023). In recent years, due to eutrophication and the rapid development of agriculture and tourism, the stability of the Erhai lakeshore ecosystem has been seriously damaged (Li et al., 2020). In 2023, Lake Erhai experienced an extreme drought, leading to the revision of the statutory minimum operating water level from 1964.30 m to 1964.10 m (DBAPPG, 2023). Although many studies have focused on the effects of water level fluctuations on aquatic plant communities in Lake Erhai (Wen et al., 2023; Wu et al., 2023; Zhu et al., 2018), there is still a lack of research on the effects of plant communities in different elevation zones along the lakeshore. Effective water level management requires a comprehensive understanding of how water level fluctuations affect plant community distribution ranges and structure in the Erhai lakeshore zones.

This study conducted comprehensive surveys and analyses of plants and soils in three elevation zones along the lakeshore of Lake Erhai during the summer and winter of 2023, and collected daily water level data provided by the Erhai Administration Bureau. The questions addressed in this study are: 1) to clarify the distribution characteristics and dynamics patterns of plant communities across different elevation zones of the lakeshore; 2) to compare summer and winter differences in the niche breadths of dominant species and their responses to elevation; and 3) to reveal the relationships among hydrology, plants, and soil, and the mechanisms influencing littoral widths. This study aims to demonstrate how seasonal water level fluctuations affect plant diversity and littoral widths in the lakeshore zone, providing a theoretical basis for effective water level management to improve the total plant diversity of the Lake Erhai.

## 2 Materials and methods

### 2.1 Study site

**Figure 1:**
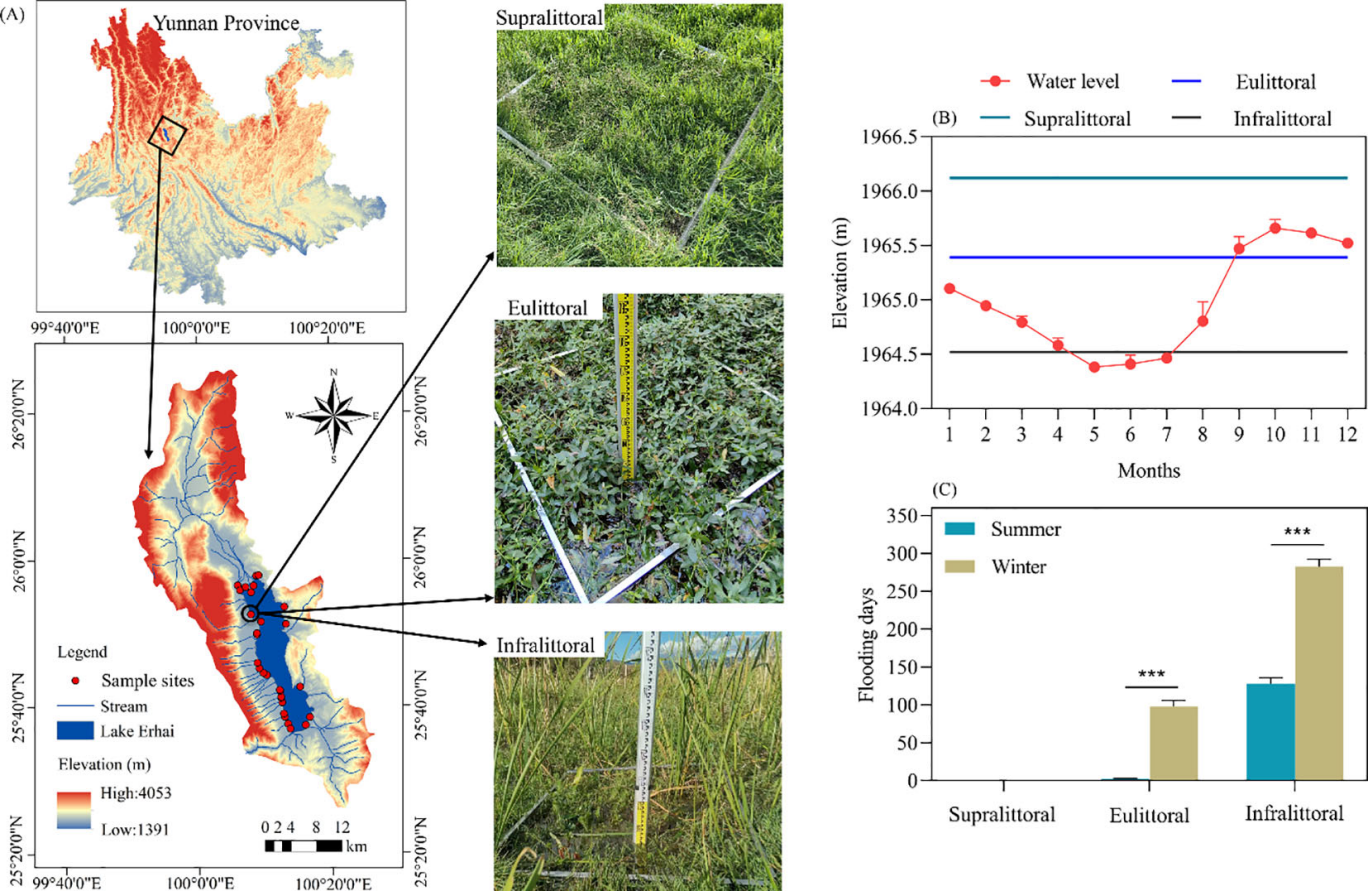
**(A)** Map of the study area and distribution of sample sites. **(B)** The monthly average water level of Lake Erhai in 2023 and the average elevations of the three elevation zones. **(C)** The difference in flooding days between summer and winter at three elevation zones. Values are means ± standard error. The significant differences are indicated by *** *p* < 0.001.

Lake Erhai (25°36′~25°58′ N, 100°06′~100°18′ E), located in Yunnan Province, China, is a faulted freshwater lake formed by crustal movement (**Figure 1**). The lake covers an area of 252 km², with average annual temperatures of 15.1°C. It has distinct wet and dry seasons, but precipitation is unevenly distributed, with more than 85% occurring during the rainy season from May to October (approximately 870 mm). A total of 117 tributaries flow into Lake Erhai, with legally authorized maximum and minimum operating water levels of 1966.0 m and 1964.3 m, respectively (Gong et al., 2023). Under the combined influence of reduced precipitation and artificial regulation since 2004, the time lag between water level and precipitation in Lake Erhai has become longer, with longer time intervals between the highest and lowest water levels, consequently altering the inundation time in the lakeshore zone (Wen et al., 2021). Lake Erhai is an important ecological area in China, characterized by high plant diversity and coverage, and rich biodiversity. The Cangshan and Erhai Nature Reserves were upgraded to national nature reserve in 1993 (Wang et al., 2023).

### 2.2 Plant surveys and soil sampling

This study conducted plant surveys and soil sampling along the lakeshore zone of Lake Erhai in the summer (mid-July) and winter (mid-December) of 2023. We selected 29 fixed sample sites based on their accessibility and the distinct, high coverage of dominant plant communities. Each site was vertically divided into three zones based on the number of days of submergence: supralittoral (1965.77m-1967.37m), eulittoral (1965.06m-1965.74m), and infralittoral (1964.30m-1965.06m). In 2023, the average number of flooding days was 0 in the supralittoral, 98 in the eulittoral, and 283 in the infralittoral (**Figure 1C**). Three parallel quadrats (1m×1m) were randomly set up in each zone, and plant name, height, coverage, biomass (fresh weight), and numbers were recorded within each quadrat. The geographical coordinates of each quadrat were recorded using a portable GPS locator and combined with spray paint and red string markers for reference.

After harvesting the aboveground biomass in the quadrats using a sickle, three soil cores (0-20 cm) were collected diagonally using a soil auger. The soil samples from each zone were thoroughly mixed to form a composite sample and transported back to the laboratory for physicochemical properties analysis. The relative elevation above the water surface in each zone was measured using a level instrument and combined with the water level recorded on the survey day to calculate the elevation of each zone (Shen et al., 2019).

### 2.3 Soil processing and analysis

The soil samples were divided into three sub-samples for different analyses. One sub-sample was used to determine soil water content (SW) using fresh soil; one sub-sample was refrigerated at 4°C to determine ammonium nitrogen (NH_4_^+^-N) and nitrate nitrogen (NO_3_^−^-N); and the other sub-sample was freeze-dried, grind, and preserved by passing it through a 0.149-mm nylon sieve to determine total phosphorus (TP), C/N ratio, total nitrogen (TN), soil organic matter (SOM), and pH.

SW was determined using the thermostat drying method: dried at 105°C for 24 hours. pH was measured using a pH meter in a mixture with a soil-water ratio of 1:2.5. NH_4_^+^-N, NO_3_^−^-N and TP were determined by UV/visible spectrophotometer (UV-1900i). TN (%) and C/N ratio were determined with an elemental analyzer (Vario Macro Cube, Germany). SOM was analyzed by the potassium dichromate volumetric method under externally heated conditions (Ji, 2005).

### 2.4 Data analysis

At the species level, the seasonal variation of the top 30 dominant species were calculated based on the ordering of importance values (*IV*). At the diversity level, assessed by Patrick index (*R*), Shannon-Wiener index (*H*), Simpson Index (*D*), Pielou Evenness index (*E*), and dominance index (*λ*). The equations for all indices were calculated as follows Equations 1–6:


(1) $$\begin{array}{c} IV=\left(\text{relative coverage}+\text{relative height}\right)\\ \left(+\text{relative biomass}+\text{relative frequency}\right)/4 \end{array}$$

(2) R=S

(3) $$H=-\sum_{i=1}^{s}P_{i}lnP_{i}$$

(4) $$D=1-\sum_{i=1}^{s}P_{i}^{2}$$

(5) $$E=\frac{H}{\ln(S)}$$

(6) $$\lambda =\sum_{i=1}^{s}P_{i}^{2}$$

where *S* is the total number of species (species richness) recorded in each zone and *Pi* represents the relative abundance of *i*th species in each zone.

Plant species niche breadth and littoral widths were calculated using the *Levins* method from package *spaa*. Meanwhile, Generalized Additive Models (GAMs) were constructed to understand the distribution ranges of plants, and to assess plant responses to elevation changes. GAMs are more flexible than generalized linear models, to explore nonlinear relationships between independent and dependent variables, and perform well in spatial prediction (Elith et al., 2006). GAMs were performed using the *mgcv* package, employing a Gaussian distribution and nonlinear fitting of species abundance and elevation through a smoothing function. The smoothing parameter was automatically selected by generalized cross validation (GCV), which obtained a low GCV value, indicating a good fit of the model (Guisan et al., 2002). The *spaa* and *mgcv* packages were both used for analysis in R (version 4.3.2).

Principal component analysis (PCA) was performed to assess correlations between components and identify the main components associated with littoral widths. PCA is an unsupervised method that identifies principal components capturing the maximum variance in the dataset without predefined explanatory variable relationships (Lever et al., 2017). Structural equation modeling (SEM) was employed to explore the relationships among flooding days, soil nutrients, SW, soil pH, plant diversity, plant parameters (R, biomass, coverage), and littoral widths. SEM is a multivariate statistical analysis method that allows for the simultaneous examination of both direct and indirect relationships among multiple observed variables, thereby revealing hidden structural patterns within complex systems. For SEM construction, we ensured the key assumptions of the linearity of relationships among variables and sufficient sample size were met. Model fitting was completed by removing the observed variables based on the modification indices and confirming that the key fit metrics met the thresholds.

All data tested for significance were assessed for variance homogeneity and normal distribution, and data satisfied with normal distribution were tested using the independent sample t-test; otherwise, the Mann-Whitney U-test was used. All significance tests for the data were conducted using IBM SPSS 27.0 software. PCA and mapping were performed using Origin2023b and ArcMap 10.8. SEM was constructed and analyzed using IBM SPSS Amos 28 software.

## 3 Results

### 3.1 Changes in species composition and diversity

A total of 110 species belonging to 40 families and 82 genera were recorded in the summer, with the highest species richness in the order of Poaceae (*N*=23, 20.9%), Asteraceae (*N*=16, 14.5%), Polygonaceae (*N*=7, 6.4%), and Cyperaceae (*N*=6, 5.5%). A total of 71 species belonging to 30 families and 58 genera were recorded in the winter, with the highest species richness in the order of Poaceae (*N*=18, 25.4%), Asteraceae (*N*=10, 14.1%), Polygonaceae (*N*=5, 7.0%), and Fabaceae (*N*=4, 5.6%). All species surveyed in this study were listed in **Appendix 1**.

**Figure 2:**
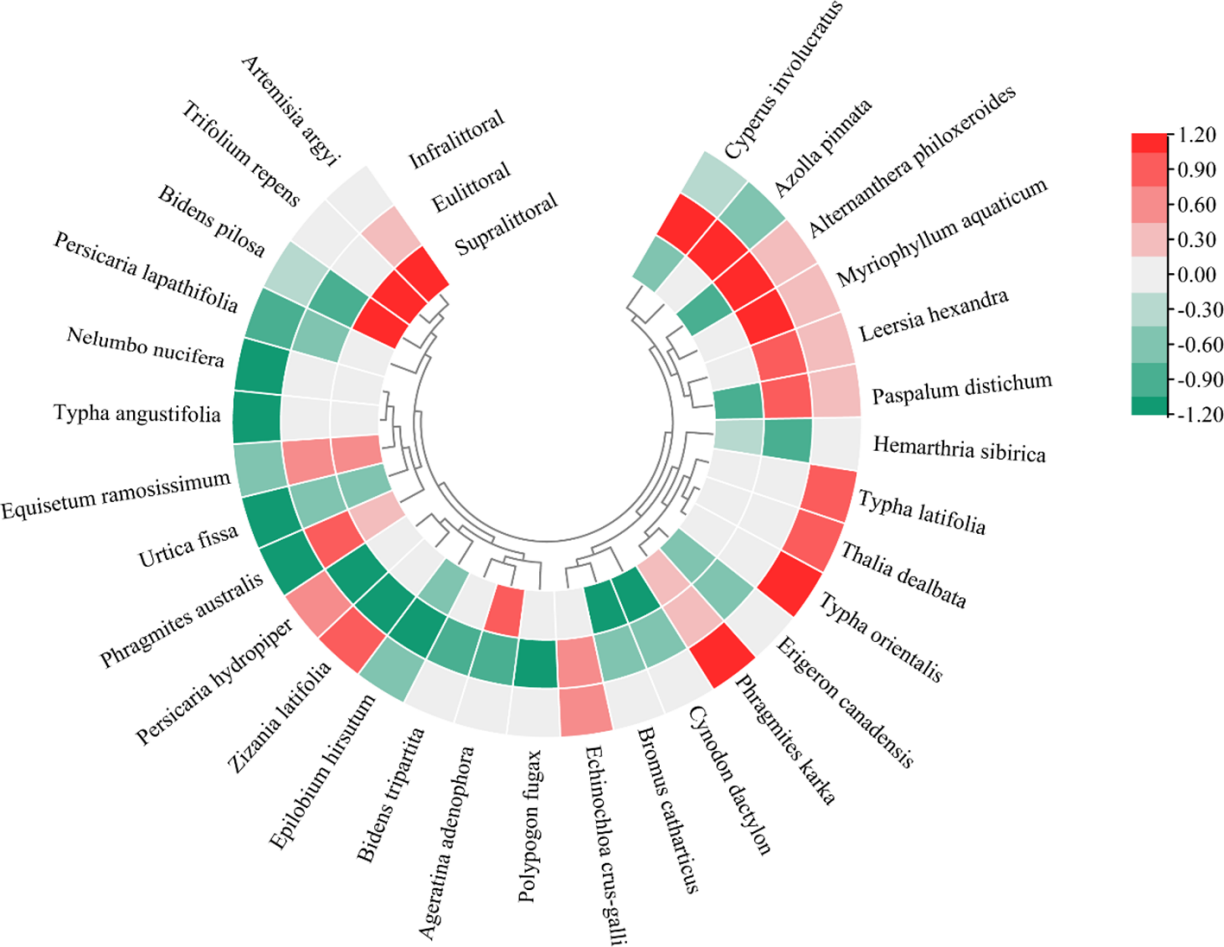
The seasonal differences in the importance values of the top 30 dominant species. Red indicates an increased value and green a decreased value.

Compared to summer, the importance values (IV) of dominant species showed seasonal differences across the three zones during winter (**Figure 2**). In the supralittoral, 7 dominant species showed an increase in IV, with *Trifolium repens* exhibiting the highest increase of 416.9%, while 10 species showed a decrease, with *Cynodon dactylon* exhibiting the highest decrease of 31.8%. In the eulittoral, 11 dominant species showed an increase in IV, with *Alternanthera philoxeroides* exhibiting the highest increase of 66.5%, while 13 species showed a decrease, with *Ageratina adenophora* exhibiting the highest decrease of 62.3%. In the infralittoral, both increased and decreased in IV were 11 dominant species, with *Phragmites karka* exhibiting the highest increase of 100% and *Phragmites australis* exhibiting the highest decrease of 40.2%.

**Figure 3:**
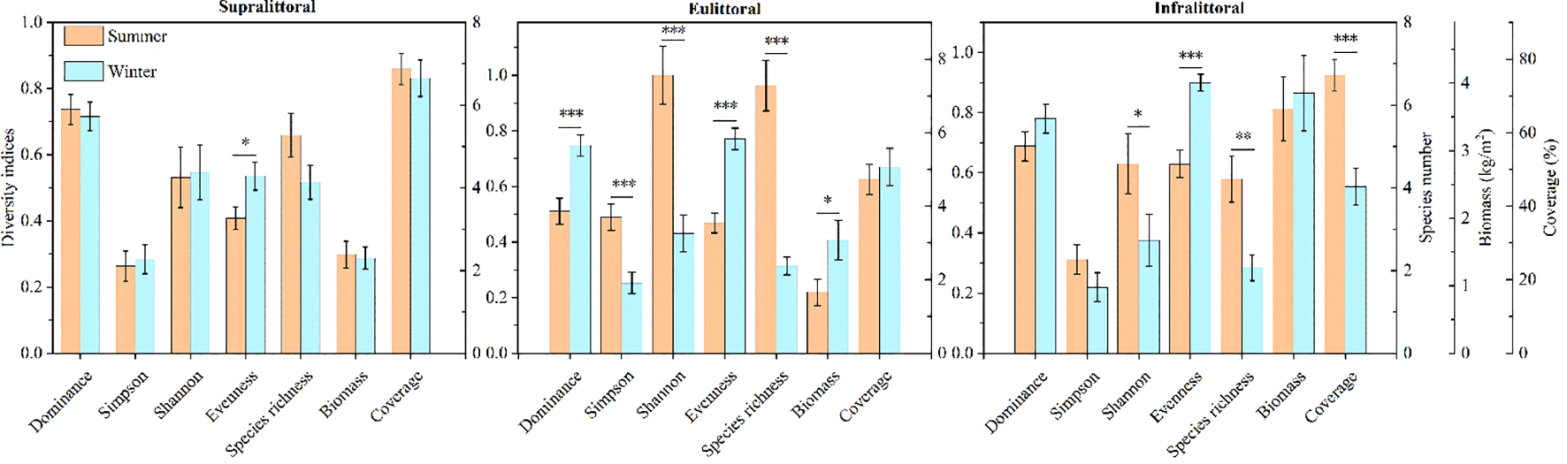
The seasonal differences in diversity indices, biomass and coverage across the three zones. Values are means ± SE. *P < 0.05, **P < 0.01, ***P < 0.001.

Compared to summer, the diversity indices showed seasonal differences across the three zones during winter (**Figure 3**). Specifically, in the supralittoral, the Evenness index increased significantly by 31.0%. In the eulittoral, the Dominance index and Evenness index increased significantly by 46.3% and 64.5%, respectively, while the Simpson index, Shannon-Wiener index, and species richness decreased significantly by 48.3%, 56.8%, and 67.3%. In the infralittoral, the Shannon-Wiener index and species richness decreased significantly by 40.4% and 50.8%, respectively, while the Evenness index increased significantly by 43.0%. In summary, seasonal differences in diversity indices were most significant in the eulittoral, followed by the infralittoral and supralittoral.

The biomass did not differ seasonally in the supralittoral and infralittoral, but increased significantly by 85.9% in the eulittoral. The coverage did not differ seasonally in the supralittoral and eulittoral, but decreased significantly by 40.1% in the infralittoral.

### 3.2 Niche breadth and species response to elevation

**Figure 4:**
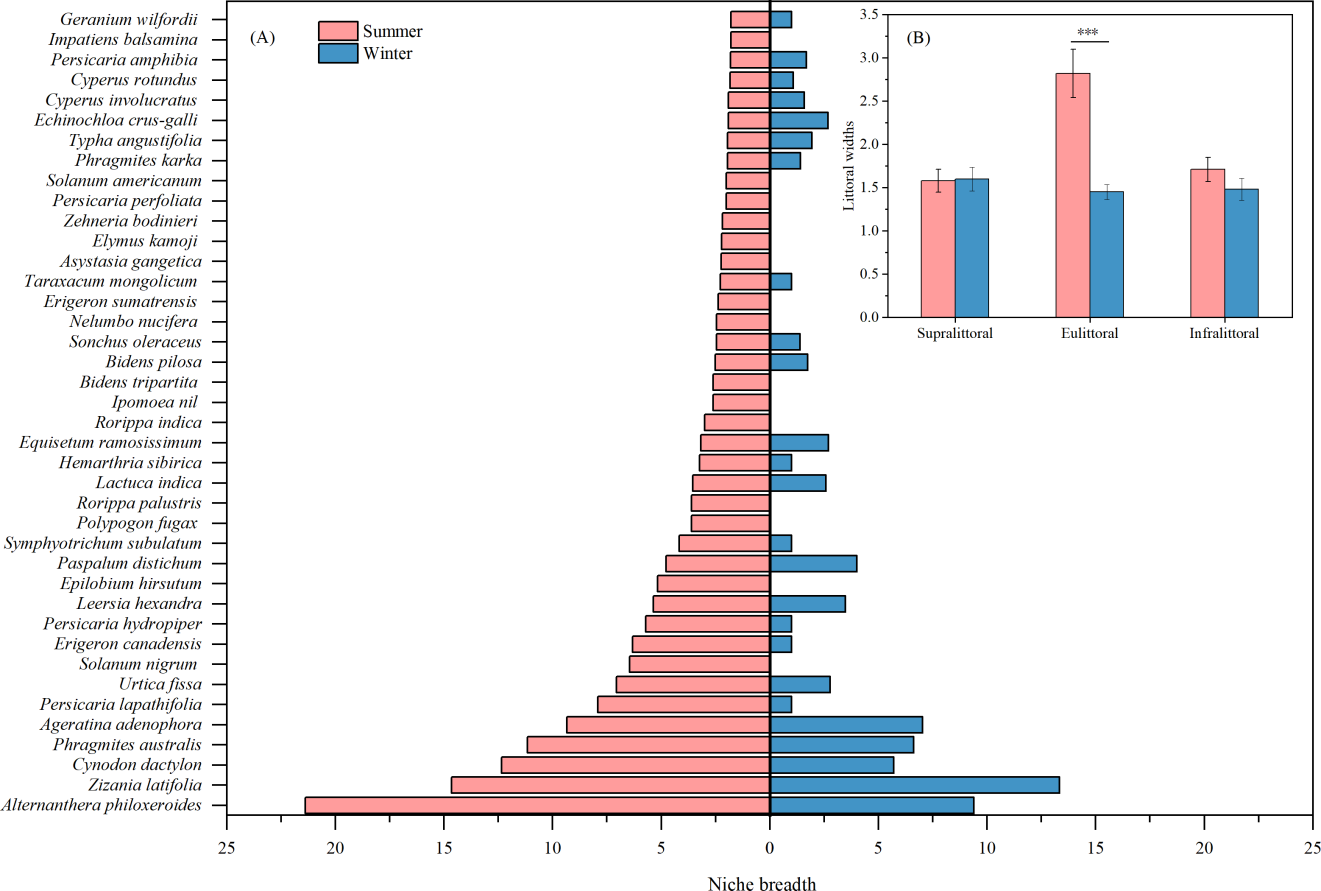
Seasonal differences in species niche breadths **(A)** and littoral widths **(B)**. Values are means ± SE. ***P < 0.001.

Seasonal comparisons of niche breadths showed significant differences among species (**Figure 4A**). The niche breadth of *Alternanthera philoxeroides* was largest in summer (21.39), but only 9.39 (decreased by 56.1%) in winter. This significant decrease indicates that rising water levels may be limiting the habitat of this species, affecting its dispersal and competitive ability. Similarly, other perennial herbaceous plants with high niche breadths in summer and significant decreases in winter include *Cynodon dactylon*, *Phragmites australis*, *Urtica fissa*, and *Epilobium hirsutum*. However, there was no significant seasonal difference in the niche breadth of the helophytes *Zizania latifolia*. This indicates that the habitat of this species is not limited by changes in water level and that it has strong adaptability. In summary, the mean niche breadth for perennial plants was 5.45 in the summer and 3.03 in the winter, showing a significant decrease of 44.4%. Most annual plants, such as *Solanum nigrum*, *Polypogon fugax*, and *Rorippa palustris*, showed a 100% decrease in niche breadth during winter.

There were no significant seasonal differences in littoral widths between the supralittoral and infralittoral (**Figure 4B**), suggesting that species composition and habitat conditions are relatively stable in both zones. However, the eulittoral exhibited a significant decrease in littoral width of 48.6% during winter (**Figure 4B**), probably caused by rising water levels that narrowed the habitat range in this zone.

**Figure 5:**
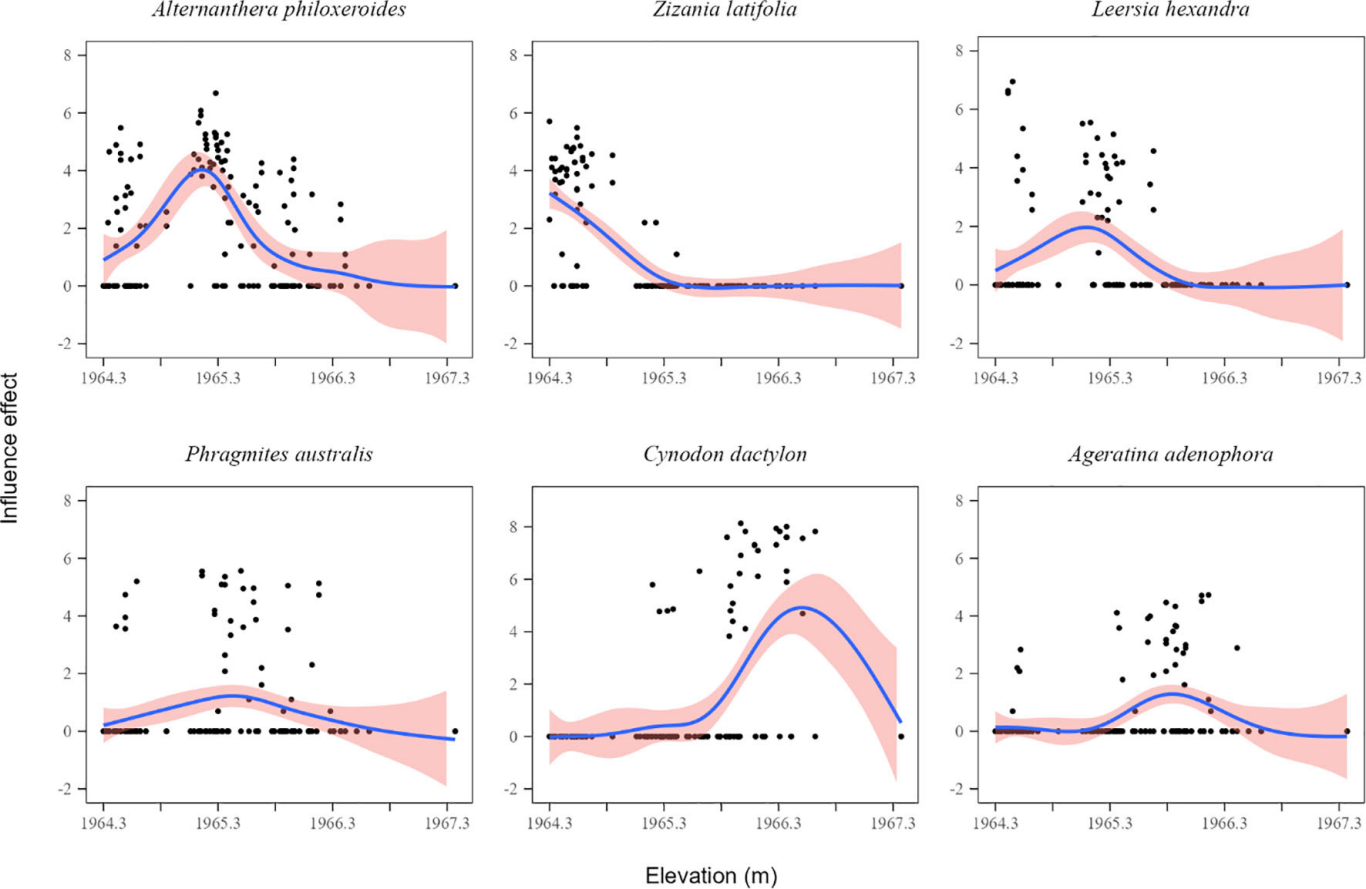
Response curves to elevation for the top 6 species. The red shaded areas indicate 95% confidence intervals.

The GAMs fitting was successful for the six dominant species (all *P* < 0.01, **Table 1**; **Figure 5**), exhibiting different elevation response curves. The response curves of *Alternanthera philoxeroides* and *Leersia hexandra* decreased with increasing elevation. Conversely, the response curve of *Cynodon dactylon* increased with increasing elevation. One species (*Zizania latifolia*) exhibited a monotonically decreasing response curve. Two species (*Phragmites australis* and *Ageratina adenophora*) exhibited symmetrical unimodal response curves.

**Table 1:** Statistical parameters of the response curves (GAMs) to elevation for the top 6 species.

Species	AIC	edf	ref.df	*F*	*P*	R^2^	GCV
*Alternanthera philoxeroides*	671.13	6.501	7.568	13.66	<0.001	0.37	2.7445
*Zizania latifolia*	561.07	4.124	5.049	37.73	<0.001	0.52	1.4565
*Leersia hexandra*	653.71	4.846	5.865	6.26	<0.001	0.17	2.4812
*Phragmites australis*	648.12	7.295	8.247	3.052	<0.01	0.11	2.4056
*Cynodon dactylon*	749.13	5.031	6.067	16.44	<0.001	0.37	4.2938
*Ageratina adenophora*	543.84	4.802	5.816	5.928	<0.001	0.16	1.3195

AIC, Akaike information criterion; edf, Estimated degree of freedom; ref.df, referenced degree of freedom; F, variance ratio; P, significance; R2, explained variance; GCV, generalized cross validation.

### 3.3 Changes in soil physico-chemical properties

**Figure 6:**
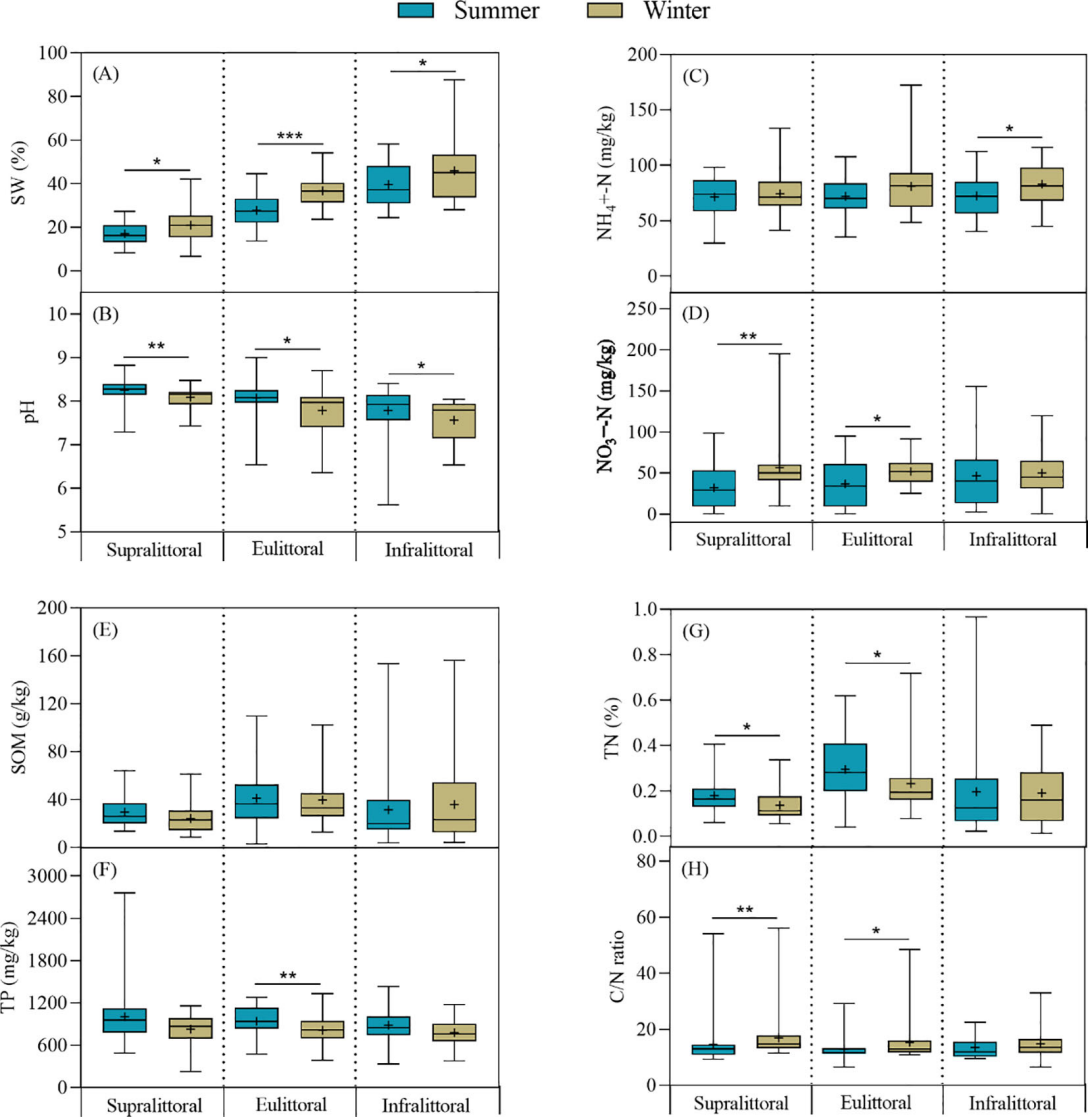
Seasonal differences in soil physico-chemical properties across three zones. **(A)** soil water content (SW), **(B)** pH, **(C)** ammonium nitrogen (NH_4_^+^-N), **(D)** nitrate nitrogen (NO_3_^−^-N), **(E)** soil organic matter (SOM), **(F)** total phosphorus (TP), **(G)** total nitrogen (TN), **(H)** C/N ratio. Values are mean ± SE, + indicates the mean. Note: * *P* < 0.05, ** *P* < 0.01, *** *P* < 0.001.

There were significant seasonal changes in soil physico-chemical properties, but the differences varied across zones during winter (**Figure 6**). Specifically, in the supralittoral, SW, NO_3_^−^-N, and the C/N ratio increased significantly, while pH and TN decreased significantly, and there were no significant differences in NH_4_^+^-N, SOM, and TP. In the eulittoral, SW, NO_3_^−^-N, and the C/N ratio increased significantly, while pH, TP, and TN decreased significantly, and there were no significant differences in NH_4_^+^-N and SOM. In the infralittoral, SW and NH_4_^+^-N increased significantly, while pH decreased significantly, and there were no significant differences in NO_3_^−^-N, SOM, TP, TN, and the C/N ratio. In summary, the seasonal differences in soil physico-chemical properties were most significant in the eulittoral, followed by the supralittoral and infralittoral. SW and pH exhibited significant seasonal differences in all three zones.

### 3.4 Relationship between littoral widths and influencing factors

**Figure 7:**
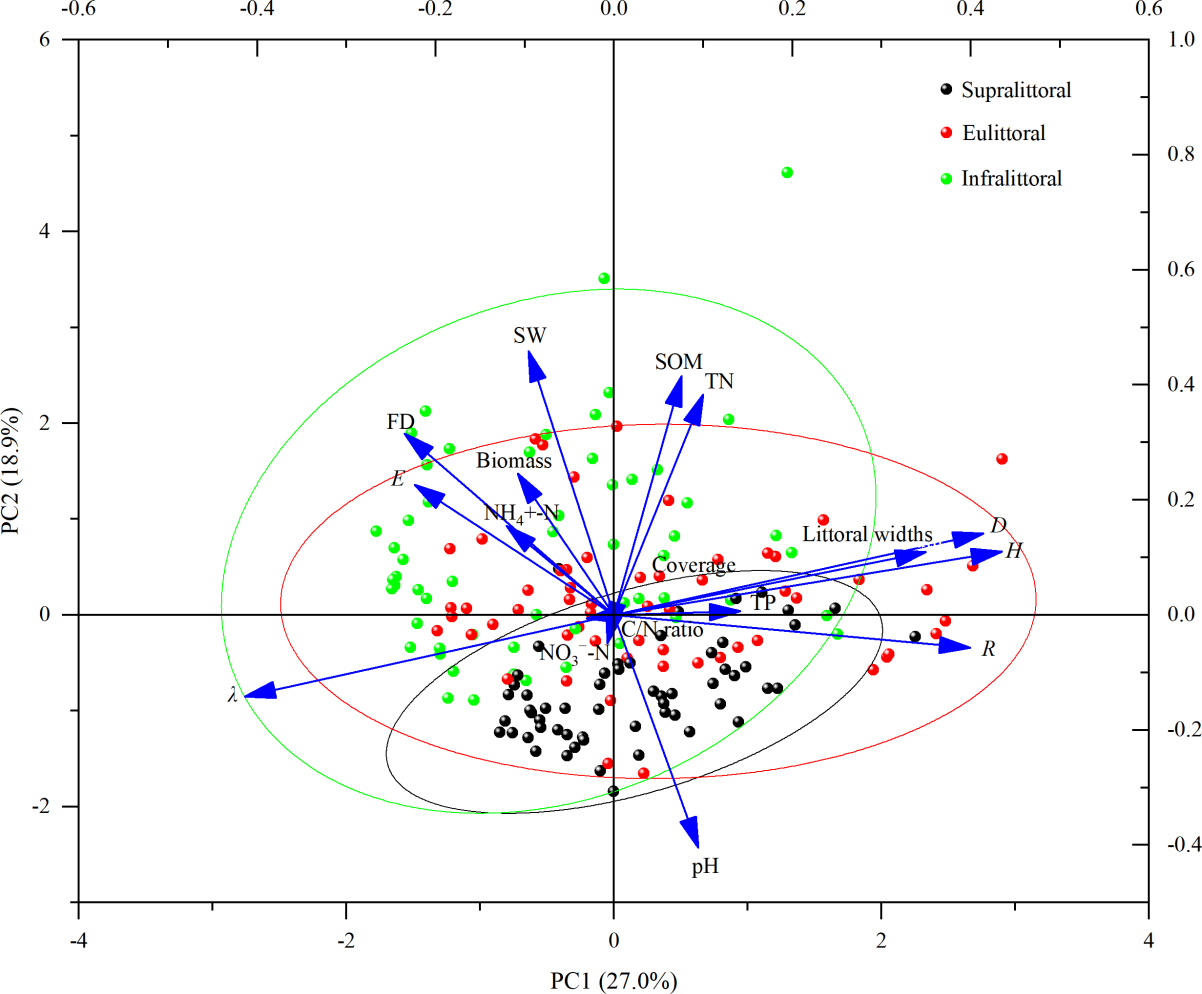
Principal component analysis of littoral widths with soil physico-chemical properties, plant diversity index, coverage, biomass, and flooding days (FD). Ellipses indicate 95% confidence intervals. SW, soil water content; NH_4_^+^-N, ammonium nitrogen; NO_3_^−^-N, nitrate nitrogen; SOM, soil organic matter; TP, total phosphorus; TN, total nitrogen; *H*, Shannon-Wiener index; *D*, Simpson Index; *E*, Pielou Evenness index; *λ*, dominance index; *R*, species richness.

Principal component analysis determined the relationship among littoral widths, soil physico-chemical properties, flooding days, plant diversity index, coverage, and biomass (**Figure 7**). The analysis results showed that the eigenvalues of the first principal component (PC1) and the second principal component (PC2) were 4.60 and 3.21, respectively, explaining 27.0% and 18.9% of the total variability. The eigenvectors of the PC1 in descending order were Shannon-Wiener index (0.43), Simpson index (0.41), species richness (0.40), TP (0.14), TN (0.10), and SOM (0.08), all of which were positively correlated with littoral widths. The eigenvectors of the PC2 in descending order were SW (0.46), FD (0.32), biomass (0.25), *E* (0.23), and NH_4_^+^-N (0.15), all of which were negatively correlated with littoral widths.

**Figure 8:**
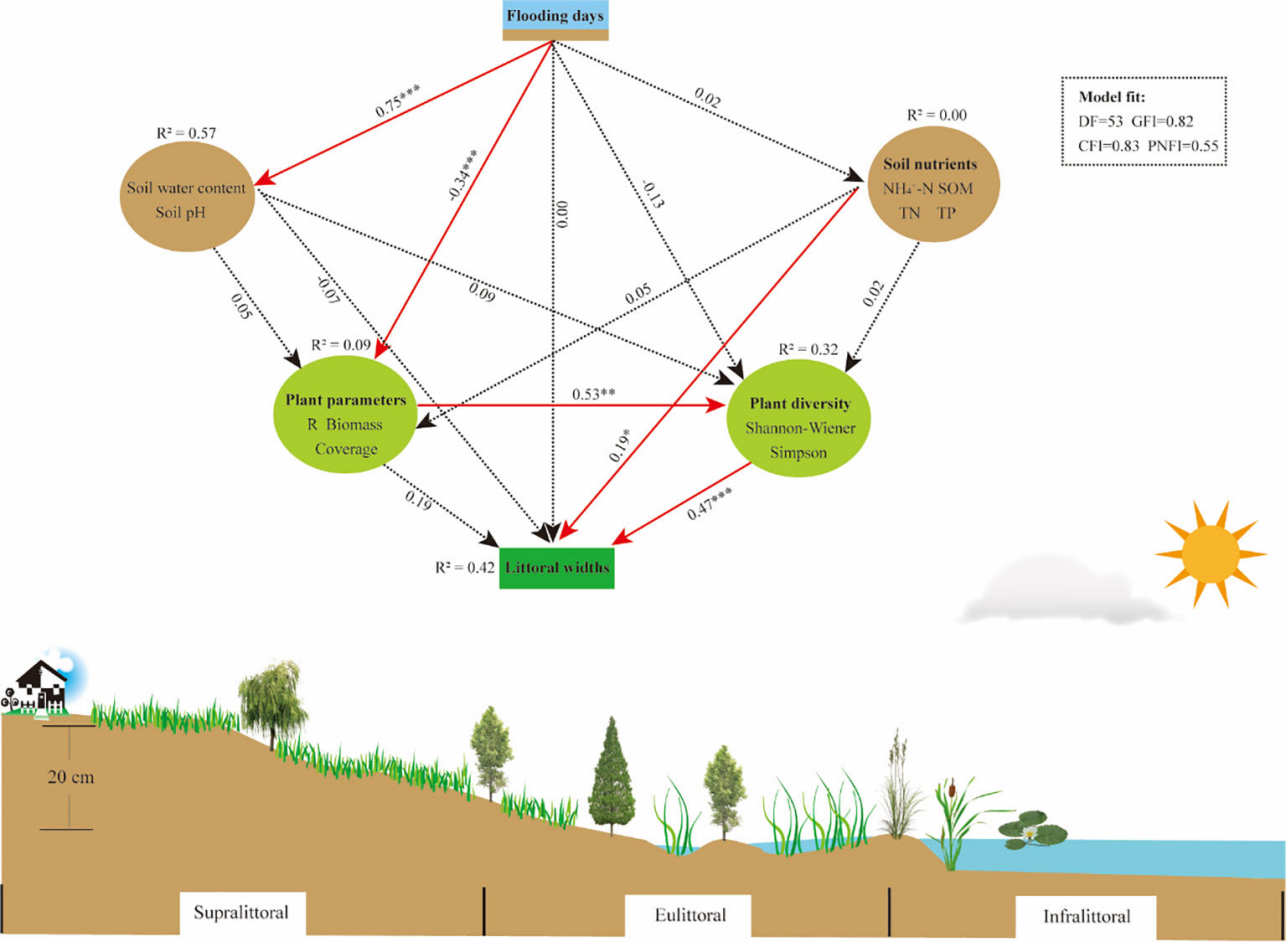
Structural equation modeling (SEM) reveals the relationship of flooding days, soil (water content, pH, nutrients), and plants (parameters, diversity) to littoral widths. Solid red arrows and dashed black arrows indicate significant and nonsignificant effects, respectively. The numbers beside the arrows represent standardized path coefficients; *P < 0.05, **P < 0.01, ***P < 0.001 indicate varying levels of significance. R^2^ values indicate the total variance explained for each variable. NH_4_^+^-N, ammonium nitrogen; SOM, soil organic matter; TN, total nitrogen; TP, total phosphorus.

Structural equation modeling path analyses revealed significant correlations among hydrology, soil, and plant variables, and that each variable was closely related to changes in littoral widths (**Figure 8**). The flooding days (FD) had a significant positive correlation with soil water content (SW) and pH, indicating that higher FD increased SW and regulated soil pH. FD had a significant negative correlation with plant parameters, indicating that changes in FD may have an important impact on plant productivity, coverage, and species richness. Moreover, plant parameters showed a significant positive correlation with plant diversity. Both soil nutrients and plant diversity exhibited a significant positive correlation with littoral widths, while FD, SW, pH, and plant parameters had no significant impact on littoral widths. In summary, FD directly affects soil (SW and pH) and plant parameters, while littoral widths were directly affected by soil nutrients and plant diversity.

## 4 Discussion

### 4.1 Effects of flooding duration on plant communities

Hydrological gradient changes directly determine the composition and range of plant communities across different elevations in the lakeshore zone (Holmquist et al., 2021; Zheng et al., 2019). We found that the importance values of hydrophytes and helophytes increased in the eulittoral and infralittoral under high water levels, while mesophytes decreased (**Figure 2**). This may be attributed to the frequent alternation of water levels in both zones, causing edge effects and changes in plant community structure and composition. Heterogeneity in lakeshore soil physicochemical properties under low-water conditions promoted microhabitat diversity, and hydrophytes and mesophytes coexisted by reducing direct competition (Zhang et al., 2022). A bigger challenge for these plants is to tolerate anoxic conditions in the substrate and supply oxygen to the roots during the high water. Hydrophytes and helophytes have well-developed root systems that efficiently absorb soil nutrients, allowing them to survive in flooded environments (Yang et al., 2024). Growth and biomass accumulation of the flood- and drought-tolerant invasive *Alternanthera philoxeroides* were promoted by adequate sunlight and water availability (Peng et al., 2021). This explains its increased importance value and biomass in the eulittoral (**Figures 2**, **3B**), with a mean biomass of 0.33 kg/m^2^ in summer and 0.79 kg/m^2^ in winter.

Significant seasonal differences were observed in diversity indices, biomass, and coverage in both the eulittoral and infralittoral, which are susceptible to water level fluctuations (**Figure 3**). Longer flooding durations, as an abiotic stressor, inhibit the survival of flood-intolerant species, thereby reducing species richness and diversity (Casanova and Brock, 2000; Huang et al., 2021). Previous studies suggest that rising water levels regulate competition in eulittoral plant communities, favoring flood-tolerant plants and driving species homogenization (Garssen et al., 2017), which is consistent with our findings. Flood-adapted species occupy broader niches, and their increased relative abundance may enhance evenness (Altenfelder et al., 2016). Supralittoral plant communities maintain stable species composition and diversity, possibly because they are unaffected by flooding (**Figure 3A**).

### 4.2 Effects of water levels changes on plant niche breadths and littoral widths

Plants adjust their niche to optimize resource use (Carscadden et al., 2020), with niche breadth being positively correlated with their adaptability and tolerance to the environment (Costa et al., 2018). Wetland plants exhibit distinct adaptive strategies across hydrological gradients, with niche breadth variations reflecting species-specific hydrologic responses (Lou et al., 2018). In this study, the niche breadth of dominant species decreased in winter (**Figure 4A**), suggesting that rising water levels reduced their resource utilization capacity. The invasive *Alternanthera philoxeroides* exhibits the highest niche breadth, suggesting it is a generalist species. However, high water levels can inhibit its regeneration and dispersal (Zhang et al., 2024). Helophytes such as *Zizania latifolia* are distributed at lower elevations, and their niche breadths are almost unaffected by water level changes (**Figures 4A**, **5**). High seed production and dispersal capacity enable *Cynodon dactylon* to quickly colonize and expand in receding environments (Li et al., 2023). Therefore, rising water levels may narrow its niche breadth.

Littoral width determines plant survival space and resource utilization, and is significantly influenced by water level changes. Rising water levels inundate low-elevation zones, driving vegetation migration upland and reducing primary distribution ranges (Leyer, 2005). Declining water levels expand plant-growing space in the lakeshore zone, increasing habitat diversity and availability (Dai et al., 2019). Our results indicated that rising water levels significantly decreased the eulittoral width but had no effect on the infralittoral and supralittoral (**Figure 4B**).

Littoral width differences are explained by seasonal variations in flooding days (**Figure 1C**): (1) Eulittoral plants are highly sensitive to periodic flooding and exposure, and prolonged inundation leads to the disappearance of flood-intolerant species (Garssen et al., 2015; Ye et al., 2020), while combined low-temperature and flooding stress restricts growth and niche breadth. (2) The supralittoral at higher elevations is less sensitive to water level fluctuations, providing stable conditions for plant survival and growth. (3) The aerenchyma and specialized leaves of helophytes and floating plants facilitate survival in hypoxic and fluctuating aquatic environments (Björn et al., 2022; Chen et al., 2002; Venter et al., 2017). Stable niche breadths were maintained by infralittoral plants despite increased flooding days, with growth likely constrained by water nutrients, water exchange, and sunlight conditions (Jin et al., 2024; Wu et al., 2023).

### 4.3 Interactive effects of flooding days, soil and plant variables on littoral widths

Seasonal variations in flooding duration caused significant changes in plants, soils, and littoral widths in the lakeshore zone. We observed that plant diversity indices were positively correlated with littoral widths (**Figures 7**, **8**), suggesting that higher plant diversity contributes to expanding niche breadth. Plant communities with high diversity reduce interspecific competition through niche differentiation, promoting efficient resource partitioning and enhancing community stability (McKane et al., 2002). Littoral width is a metric for predicting range size and is positively correlated with environmental tolerance breadth (Slatyer et al., 2013). A broader width indicates that plant communities can adapt to a wider range of environmental conditions, with high ecological resilience and competitiveness.

Subtle water level fluctuations alter wetland plant distribution and ecological processes (Liu et al., 2020; Qin et al., 2017), and similarly impact habitat conditions such as soil redox potential, microbial activity, and oxygen availability, which in turn influence plant growth and community composition (Hájek et al., 2013; Huang et al., 2023). Nutrient availability gradients alter plant diversity and productivity, influencing habitat ranges for different vegetation types (Zhang et al., 2022). For example, organic-rich soils foster aquatic plant growth (Silveira and Thomaz, 2015), while xerophytes dominate in low-nutrient soils (Fan et al., 2019). Therefore, flood duration influences littoral width by altering soil properties, plant diversity and niche breadth, and promoting plant community environmental adaptations.

### 4.4 Management implications

Our results suggest that high water levels reduced the niche breadth of most dominant perennial plants and narrowed the eulittoral width, while littoral widths were positively correlated with plant diversity and soil nutrients. Based on our findings and management needs, we recommend: 1) Regulate water levels to stabilize eulittoral habitat and promote plant diversity; 2) Optimize plant community vertical structure across elevation gradients to maintain plant diversity under water level fluctuations; 3) Develop appropriate water level thresholds to avoid extreme drought-flood events that threaten lakeshore plants.

## 5 Conclusions

Water level fluctuations not only shape the vertical structure of wetland plants, but are also a crucial factor influencing plant community composition and diversity. We investigated the seasonal variations in plant diversity, coverage, aboveground biomass, and soil physicochemical properties across three elevation ranges in the Erhai lakeshore zone, and explored the influence of hydrological gradient changes on littoral widths as well as the potential relationships. We found that the most significant seasonal differences in diversity indices, biomass, littoral widths, and soil physicochemical properties were observed in the eulittoral, followed by the infralittoral and supralittoral. Soil nutrients and plant diversity are the main influences on littoral widths. In addition, species response curves provide further insight into the elevation range of plant distribution in the lakeshore zone, which helps to capture the response of plant niche breadth to changes in the hydrologic gradient. These findings deepen our understanding that seasonal hydrologic changes affect plant communities and littoral widths in the lakeshore zone, and provide crucial guidance for developing effective wetland water level management in response to extreme drought events.

## Data Availability

The original contributions presented in the study are included in the article/supplementary material. Further inquiries can be directed to the corresponding authors.

## Appendix 1 Total list of plant surveys in the Erhai lakeshore zone during 2023.

**Table:**

	Species	Families	Genera
1	*Viola prionantha*	Violaceae	Viola
2	*Mazus pumilus*	Mazaceae	Mazus
3	*Veronica anagallis-aquatica*	Plantaginaceae	Veronica
4	*Plantago major *	Plantaginaceae	Plantago
5	*Mentha canadensis*	Lamiaceae	Mentha
6	*Clinopodium chinense*	Lamiaceae	Clinopodium
7	*Juncus effusus*	Juncaceae	Juncus
8	*Trifolium repens*	Fabaceae	Trifolium
9	*Medicago lupulina*	Fabaceae	Medicago
10	*Vicia sepium*	Fabaceae	Vicia
11	*Vicia sativa*	Fabaceae	Vicia
12	*Melilotus albus*	Fabaceae	Melilotus
13	*Impatiens balsamina*	Balsaminaceae	Impatiens
14	*Echinochloa crus-galli*	Poaceae	Echinochloa
15	*Polypogon fugax*	Poaceae	Polypogon
16	*Bromus catharticus *	Poaceae	Bromus
17	*Elymus kamoji*	Poaceae	Elymus
18	*Cynodon dactylon*	Poaceae	Cynodon
19	*Zizania latifolia*	Poaceae	Zizania
20	*Lolium perenne *	Poaceae	Lolium
21	*Eulalia speciosa*	Poaceae	Eulalia
22	*Arthraxon hispidus*	Poaceae	Arthraxon
23	*Phragmites karka*	Poaceae	Phragmites
24	*Phragmites australis*	Poaceae	Phragmites
25	*Leersia hexandra*	Poaceae	Leersia
26	*Paspalum dilatatum*	Poaceae	Paspalum
27	*Paspalum distichum*	Poaceae	Paspalum
28	*Hemarthria altissima*	Poaceae	Hemarthria
29	*Rottboellia cochinchinensis*	Poaceae	Rottboellia
30	*Coix lacryma-jobi*	Poaceae	Coix
31	*Poa annua*	Poaceae	Poa
32	*Setaria palmifolia*	Poaceae	Setaria
33	*Setaria pumila*	Poaceae	Setaria
34	*Setaria faberi*	Poaceae	Setaria
35	*Chloris virgata*	Poaceae	Chloris
36	*Capillipedium parviflorum *	Poaceae	Capillipedium
37	*Actinostemma tenerum*	Cucurbitaceae	Actinostemma
38	*Zehneria bodinieri *	Cucurbitaceae	Zehneria
39	*Azolla pinnata subsp. asiatica*	Salviniaceae	Azolla
40	*Malva verticillate var. crispa*	Malvaceae	Malva
41	*Sida acuta*	Malvaceae	Sida
42	*Artemisia argyi*	Asteraceae	Artemisia
43	*Artemisia selengensis*	Asteraceae	Artemisia
44	*Lactuca indica*	Asteraceae	Lactuca
45	*Bidens pilosa*	Asteraceae	Bidens
46	*Bidens tripartita*	Asteraceae	Bidens
47	*Erigeron canadensis*	Asteraceae	Erigeron
48	*Sonchus oleraceus*	Asteraceae	Sonchus
49	*Galinsoga parviflora*	Asteraceae	Galinsoga
50	*Taraxacum mongolicum*	Asteraceae	Taraxacum
51	*Senecio scandens*	Asteraceae	Senecio
52	*Pseudognaphalium affine*	Asteraceae	Pseudognaphalium
53	*Erigeron sumatrensis*	Asteraceae	Erigeron
54	*Artemisia indica*	Asteraceae	Artemisia
55	*Crassocephalum crepidioides*	Asteraceae	Crassocephalum
56	*Ageratina adenophora*	Asteraceae	Ageratina
57	*Symphyotrichum subulatum*	Asteraceae	Symphyotrichum
58	*Sonchus asper*	Asteraceae	Sonchus
59	*Youngia japonica*	Asteraceae	Youngia
60	*Asystasia gangetica*	Acanthaceae	Asystasia
61	*Hypoestes triflora*	Acanthaceae	Hypoestes
62	*Nelumbo nucifera*	Nelumbonaceae	Nelumbo
63	*Persicaria japonica*	Polygonaceae	Persicaria
64	*Persicaria perfoliata*	Polygonaceae	Persicaria
65	*Persicaria amphibia*	Polygonaceae	Persicaria
66	*Persicaria hydropiper*	Polygonaceae	Persicaria
67	*Persicaria lapathifolia*	Polygonaceae	Persicaria
68	*Persicaria longiseta*	Polygonaceae	Persicaria
69	*Rumex acetosa*	Polygonaceae	Rumex
70	*Rumex dentatus*	Polygonaceae	Rumex
71	*Rumex hastatus*	Polygonaceae	Rumex
72	*Oenothera rosea*	Onagraceae	Oenothera
73	*Epilobium hirsutum*	Onagraceae	Epilobium
74	*Verbena officinalis*	Verbenaceae	Verbena
75	*Geranium wilfordii *	Geraniaceae	Geranium
76	*Clematis florida *	Ranunculaceae	Clematis
77	*Canna × generalis*	Cannaceae	Canna
78	*Canna indica*	Cannaceae	Canna
79	*Equisetum ramosissimum*	Equisetaceae	Equisetum
80	*Equisetum arvense*	Equisetaceae	Equisetum
81	*Rubia cordifolia*	Rubiaceae	Rubia
82	*Potentilla reptans*	Rosaceae	Potentilla
83	*Duchesnea indica*	Rosaceae	Duchesnea
84	*Solanum nigrum*	Solanaceae	Solanum
85	*Solanum americanum*	Solanaceae	Solanum
86	*Torilis scabra*	Apiaceae	Torilis
87	*Oenanthe javanica*	Apiaceae	Oenanthe
88	*Cyperus involucratus*	Cyperaceae	Cyperus
89	*Cyperus fuscus*	Cyperaceae	Cyperus
90	*Cyperus rotundus*	Cyperaceae	Cyperus
91	*Cyperus michelianus*	Cyperaceae	Cyperus
92	*Fimbristylis dichotoma*	Cyperaceae	Fimbristylis
93	*Phytolacca acinosa*	Phytolaccaceae	Phytolacca
94	*Rorippa indica*	Brassicaceae	Rorippa
95	*Rorippa globosa*	Brassicaceae	Rorippa
96	*Rorippa palustris*	Brassicaceae	Rorippa
97	*Capsella bursa-pastoris*	Brassicaceae	Capsella
98	*Stellaria aquatica*	Caryophyllaceae	Stellaria
99	*Nymphoides peltata*	Menyanthaceae	Nymphoides
100	*Colocasia antiquorum*	Araceae	Colocasia
101	*Lemna minor*	Araceae	Lemna
102	*Amaranthus blitum*	Amaranthaceae	Amaranthus
103	*Chenopodium album*	Amaranthaceae	Chenopodium
104	*Alternanthera philoxeroides*	Amaranthaceae	Alternanthera
105	*Typha latifolia*	Typhaceae	Typha
106	*Typha angustifolia*	Typhaceae	Typha
107	*Typha orientalis*	Typhaceae	Typha
108	*Myriophyllum aquaticum*	Haloragaceae	Myriophyllum
109	*Myriophyllum spicatum*	Haloragaceae	Myriophyllum
110	*Ceratophyllum demersum*	Ceratophyllaceae	Ceratophyllum
111	*Calystegia hederacea *	Convolvulaceae	Calystegi
112	*Ipomoea nil*	Convolvulaceae	Ipomoea
113	*Convolvulus arvensis*	Convolvulaceae	Convolvulus
114	*Urtica atrichocaulis*	Urticaceae	Urtica
115	*Urtica fissa*	Urticaceae	Urtica
116	*Commelina communis*	Commelinaceae	Commelina
117	*Potamogeton wrightii*	Potamogetonaceae	Potamogeton
118	*Potamogeton maackianus*	Potamogetonaceae	Potamogeton
119	*Thalia dealbata*	Marantaceae	Thalia
120	*Oxalis corniculata*	Oxalidaceae	Oxalis
121	*Sagittaria trifolia*	Alismataceae	Sagittaria
122	*Vallisneria natans*	Hydrocharitaceae	Vallisneria
123	*Euphorbia esula *	Euphorbiaceae	Euphorbia
124	*Pontederia crassipes*	Pontederiaceae	Pontederia
